# Artemisinin and Its Derivatives as a Repurposing Anticancer Agent: What Else Do We Need to Do?

**DOI:** 10.3390/molecules21101331

**Published:** 2016-10-07

**Authors:** Zhe Li, Qin Li, Jun Wu, Manyuan Wang, Junxian Yu

**Affiliations:** 1 Department of Pharmacy, Beijing Friendship Hospital, Capital Medical University, Beijing 100050, China; jamesbangzhe@sina.com; 2 Cancer Center, Beijing Friendship Hospital, Capital Medical University, Beijing 100050, China; qinli128003@163.com (Q.L.); woshiwujun0613@sina.com (J.W.); 3 School of Traditional Chinese Medicine, Capital Medical University, Beijing 100069, China; wangmy@ccmu.edu.cn

**Keywords:** artemisinin, anticancer activity, repurposed, specific drug delivery system, clinical trials

## Abstract

Preclinical investigation and clinical experience have provided evidence on the potential anticancer effect of artemisinin and its derivatives (ARTs) in the recent two decades. The major mechanisms of action of ARTs may be due to toxic-free radicals generated by an endoperoxide moiety, cell cycle arrest, induction of apoptosis, and inhibition of tumor angiogenesis. It is very promising that ARTs are expected to be a new class of antitumor drugs of wide spectrum due to their detailed information regarding efficacy and safety. For developing repurposed drugs, many other characteristics of ARTs should be studied, including through further investigations on possible new pathways of anticancer effects, exploration on efficient and specific drug delivery systems-especially crossing biological barriers, and obtaining sufficient data in clinical trials. The aim of this review is to highlight these achievements and propose the potential strategies to develop ARTs as a new class of cancer therapeutic agents.

## 1. Introduction

Only one of every 5000–10,000 prospective anticancer agents are approved by the Food and Drug Administration (FDA) and only 5% of oncology drugs that enter Phase I clinical trials are ultimately approved. These failure rates underscore the need for alternative efforts for drug development. Some old approved drugs, whose detailed information about the drug formulation and safety are usually available, may hold promise to have much faster clinical trials than a brand-new drug [1]. Aspirin, a famous nonsteroidal anti-inflammatory drug (NSAID), was proved to not only be useful in reducing short-term and long-term cancer incidence and mortality both in men and women, but also be effective in reducing the growth and metastasis of malignancy as well [2,3,4,5]. Metformin, an antidiabetic drug, is a potent 5′ AMP-activated protein kinase (AMPK)-independent antiproliferative agent and associated with the decreased risk of the occurrence of cancers [6,7]. Other good examples are also included thalidomide and cimetidine [8,9,10,11]. Beyond western medicines mentioned above, a few traditional Chinese medicines are also repurposed as a treatment for cancers; for instance, arsenic. Arsenic trioxide, a highly toxic medicine that is used to treat lung diseases and psoriasis at very low doses, showed outstanding curative effects in clinical cases with acute promyelocytic leukemia [12,13] (Table 1).

Beyond those mentioned above, another promising repurposing agent for anticancer activity are artemisinin and its derivatives (ARTs). Artemisinin, primarily used for the treatment of malaria cases since 1973 [14], is a most effective weapon against malarial parasites, especially for falciparum malaria and cerebral malaria, clinically [15,16]. In addition, recent studies also demonstrated that artemisinin not only has antiparasitic effects, including against plasmodium and schistosome, but also has significant anti-inflammatory and immunomodulatory actions [17]. Besides all that, another very impressive attention of ART is the potential anticancer effect that was revealed in the recent two decades. In the 1990s, Woerdenbag et al. firstly reported the anticancer effects of artemisinin [18]. The experiments in vitro and in vivo showed that artemisinin and its derivatives can inhibit and kill a variety of tumor cells. The results of the experiment in vitro conducted by the U.S. National Cancer Institute (NCI) in 55 kinds of tumor cell lines showed that artesunate had inhibitory effect on the cells of leukemia, colorectal cancer, melanoma, breast cancer, ovarian cancer, prostate cancer, kidney cancer, etc. [19]. In the previous study, we also found that artemisinin and dihydroartemisinin exhibit significant anticancer effects against human hepatoma cells with minimal effects on normal cells, regardless of p53 status, and that the latter was also effective in ovarian cancer when administered alone or in combination with carboplatin [20,21]. Similarly, it was proven that artemether and arteether had antitumor activities in some malignant cells, such as gastric cancer, glioma, and breast cancer [22,23,24]. Artemisinins have been brought by NCI into a program of antitumor drug screening and activity research. More concerning, however, is that artemisinin was being considered for transformation from research study to clinic use. A clinical trial in Germany was conducted to observe artesunate and dihydroartemisinin when they were orally administrated with artemisinin and artesunate to patients with metastatic breast cancer [25].

There is growing evidence that ARTs are expected to be a class of new antitumor drugs of wide spectrum. This review highlights achievements in artemisinin and its derivatives, initially well-characterized antimalarials, mainly for their investigations and uses in oncology. In addition to outlining the advantages of repurposing, the potential and strategies to develop ART analogs as cancer therapeutic agents are also discussed.

## 2. Foregoing Researches on the Anticancer Effect of Artemisinin and Its Derivates

### 2.1. Chemistry of Artemisinin and Its Derivates

**Figure 1:**
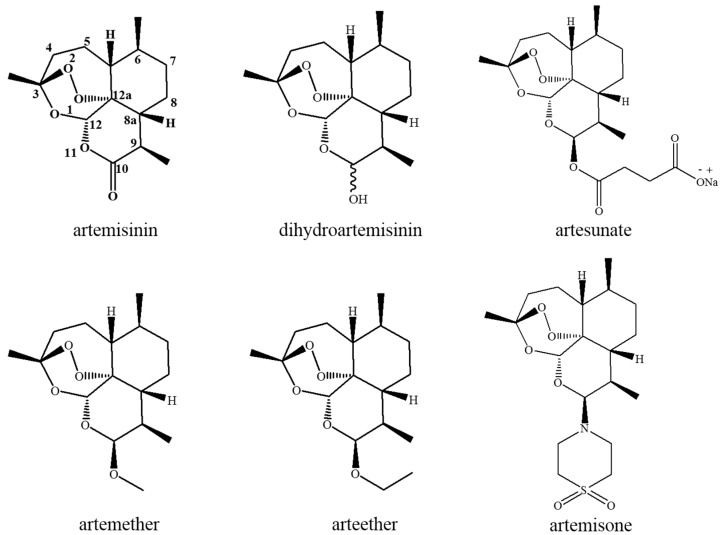
Artemisinin and its derivatives with anticancer activity.

Artemisinin (ART), isolated and extracted from dried leaves and flower buds of *Haplopappus gracilis Artemisia annua* L., is a sesquiterpene lactone compound containing an endoperoxide radical without nitrogen atoms in its structure [14,26]. *Artemisia annua* L. is a complex compound containing more than 200 components that have been identified to date. Its chemical composition can be divided into the volatile and nonvolatile classes. ART only exists in the *Artemisia annua* L., and its content is greatly associated with its origin. There is a lower level of ART in the *Artemisia annua* L. that is produced in Europe, America, and northern and eastern China. Even in the *Artemisia annua* L. that isproduced in southwest China, the content of ART is only 0.6%–1.0%. Other nonvolatile components in *Artemisia annua* L. also include arteannuin A, arteannuin B, arteannuin C, artemisinic acid, artemisia methyl ester, artemisinol, artemisilactone, etc., all of which are sesquiterpene lactones [15,27]. Since the advent of ART, its derivatives such as dihydroartemisinin, artesunate, artemether, and arteether have been investigated and successively obtained around the world. Among them, the last two are rapidly metabolized to dihydroartemisinin after entering the body. These compounds have proved to have significant antitumor activities as well. Other artemisinin-derivate monomers that were developed for antimalarials—artemisone, arterolane, artemiside, artelinic acid, and artesunic acid—had almost no effects against cancer cells, except for artemisone [28] (Figure 1). The main active anticancer sites of ART are the same astheir antimalarial sites. Although all anticancer abilities cannot be removed completely due to a lack of peroxide bridges, the inhibitory effect of tumor cells is greatly reduced [29]. Therefore, all structural modification is conducted on the basis of keeping the peroxide bridge structure, focusing mainly on the 9,10-portion of modification, especially the most common 10-portion of modification. In addition to the basic monomeric compounds, new classes of artemisinin-like dimers with antitumor activity have been developed [30].

### 2.2. Known Anticancer Activity and Mechanism of Action

How do ARTs kill tumor cells? It has been found that cell membranes were the principal target sites. ARTs can not only induce apoptosis, but also play a role in cell swelling. Once cytomembranes are destroyed, membrane permeability is altered, thus resulting in the death of cells. Furthermore, ARTs can also induce apoptosis; inhibit angiogenesis, proliferation, and migration of vascular endothelial cells; and improve chemotherapy or radiotherapy sensitization.

#### 2.2.1. Toxic-Free Radicals Generated by Endoperoxide Moiety

**Figure 2:**
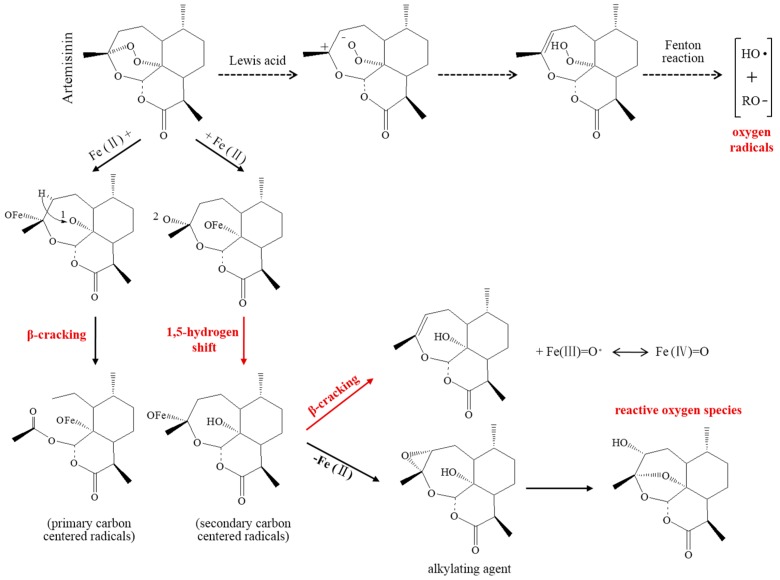
Speculated mechanisms of artemisinin generating oxidative damage via ferrous ion-mediated reactions: the β-cracking and 1,5-hydrogenmigration are primary pathways, and the Lewis is a secondary pathway.

Although the exact mechanisms of action of ART derivatives has not been fully elucidated yet, the C-radical hypothesis has become one of the most widely accepted theories. Throughout the whole process, the toxic-free radicals generated by the endoperoxide moiety of ART via a ferrous iron-mediated reaction are essential to killing or attenuating tumor cells (Figure 2). The peroxide bridge of ART analogs can react with the ferrous atom and produce free radicals or reactive oxygen species (ROS) using the carbon as the core. ROS play a very important role in killing specific tumor cells, for example, by inducing apoptosis and oxidative DNA damage [31,32,33]. Due to the lack of antioxidant enzymes, the tumor cells are more susceptible to damage caused by ROS, and strong oxidative stress is the universal mechanism of anticancer drugs. A recent study shows that HeLa cells, after treatment with artesunate, can generate ROS earlier than when the cytotoxic effects occur, thus indicating that the role of ROS may be one of initiating factors in triggering cell damage [34]. Compared with normal cells, cancer cells need a lot of iron involved inactive nucleic acid metabolism, while the majority of the surface of the tumor cells has a high density of transferrin receptor (TfR). Tumor cells with a high expression of TfR are more susceptible to ART-based drugs [35]; the iron-rich cancer cells can stimulate cytotoxic effects of ARTs. After coincubation with the amino acetic ferrous sulfate, these ART derivatives can significantly improve the sensitivity of leukemia CCRF-CEM cells and human astrocytoma U373 cells to ART [36]. Moreover, after pretreatment with the iron complexant deferoxamine mesylate, leukemia cells can antagonize dihydroartemisinin-induced apoptosis [37].

#### 2.2.2. Cell Cycle Arrest

ART derivatives can inhibit the proliferation of tumor cells by affecting the cell cycle at any stage, with the G0/G1 to S phase inhibition most commonly seen, and its mechanism of action is achieved by changing expression and activity of regulatory enzymes in the cell cycle [38,39]. Dihydroartemisinin-induced cell cycle arrest at the G0/G1 phase can occur through the down regulation of cyclins and the transcriptional activity of cyclin-dependent kinases (CDKs) by inhibiting the activity of the CDK promoter or enhancing the activity of CDK inhibitors [20]. For example, ART can directly inhibit CDK-4 gene expression, leading to cell cycle arrest [40]. Artesunate induces cell cycle arrest in the G2/M phase by up regulating the expression of Beclin1, which is an initiator of autophagy [41]. In addition, artesunate may interfere with some genes such as Bub3, Mad3, and Mad2, which are regulators of the mitotic spindle checkpoint in the G2/Mphase [42]. A study also found that the sensitivity of ART compounds was highly correlated to the cell division cycle 25homolog A (CDC25A) proteins. CDC25A genes that are transferred into rat embryonic R12 cells may improve their sensitivity to artesunate [43]. Dihydroartemisinin can prevent the proliferation of pancreatic cancer cells by inhibiting the activity of nuclear transcription factor NF-κB, and inhibit the proliferation of lung cancer cells by down regulating the surviving protein (a protein which can regulate apoptosis and cell cycle G2/M phase) [43].

#### 2.2.3. Induction of Apoptosis

The apoptosis processes can be regulated through the proapoptotic genes and antiapoptotic genes, and the effects of these two types of genes on the mitochondrion. The role of ARTs is to inhibit proliferation and induce apoptosis rather than promoting necrocytosis, and it has more efficiency with the help of iron transporters [44,45,46,47]. Generally, ART compounds can induce apoptosis through mitochondria-mediated pathways [48,49]. ART derivates can play their roles in proapoptosis by not only promoting the release of cytochrome 3 and the overexpression of Bax, but also by increasing the Bax/Bcl-2 ratio and activating caspase-3 and caspase-9 inosteosarcoma cells [50,51]. Dihydroartemisinin can effectively improve the cell proapoptotic protein Bax and down regulate the antiapoptotic protein Bcl-2 while increasing Fas and activating caspase-8, and inhibit translocation and DNA-binding activity of nuclear factor-κappa gene binding (NF-κB) as well [43,52,53]. Dihydroartemisinin sensitivity was positively correlated with the expression levels of c-Myc oncoprotein, and the tumor cells with high expression of c-Myc were more sensitive to the compound [54]. It is also worth mentioning that the apoptosis-induction effects of dihydroartemisinin were independent of p53 status (either wild-type or mutant-type) [20]. Another probable mechanism of action of ARTs on tumor cells is due to their direct interaction with DNA replication [55]. Some researchers confirm that artemisinin and it derivatives display the ability of inhibiting certain viral propagation such as cytomegalovirus, herpesvirus, and hepatitis virus [56,57]. The antiviral mechanism of ARTs is precisely because of DNA interference. ART analogs that also stimulated increases in intracellular calcium levels and triggered oxidative damage may also be involved in apoptosisin tumor cells [58,59,60]. In addition, the latest research demonstrates that dihydroartemisin in participates in the process of autophagy, which is closely related to programmed cell death [61].

#### 2.2.4. Inhibition of Tumor Angiogenesis

Neovascularization plays a key role in progression and metastasis of malignant tumors and are regulated by a variety of active substances named proangiogenic and antiangiogenesis factors. The former includes a large class of growth factors or cytokines such as Vascular endothelial growth factor (VEGF), Basic Fibroblast Growth Factor (bFGF), angiopoietin, Matrix metalloproteinases (MMPs), interleukin-1 (IL-1), IL-8, and the latter includes endostain, angiostain, Tissue inhibitor of matrix metalloproteinases (TIMPs), etc. ARTs can inhibit the formation of tumor blood vessels by downregulating the production of growth factors as well as upregulating the inhibitory factors [62]. Among artemisinin derivatives, dihydroartemisinin and artesunate, especially the latter, are the most desired. Data suggested that dihydroartemisin in inhibited angiogenesis largely relying on the transcription factor NF-κB pathway. It can repress the binding of NF-κB to DNA and down regulate the proangiogenic factors such as VEGF, Vascular Endothelial Growth Factor Receptor 2 (VEGFR2), IL-8, Cyclooxygenase-2 (COX-2) and MMP9 [63,64,65,66]. In recent decades, more attention was paid gradually to the inhibitory effects of artesunate on angiogenesis [67]. The target molecules for the response of tumor cells to artesunate consist of EGF, VEGF, VEGFR, and so on [68,69]. Moreover, inhibitory effects of artesunate on VEGF expression are correlated closely with the level secreted by cells. In addition to the above mechanism, other targets are also found such as β-catenin, E-cadherin, tumor growth factor β2, c-Src tyrosine kinase, and breast cancer susceptibility gene 2 (BRCA2) [70]. With further research, it was verified that artesunate had a synergistic inhibition of angiogenesis [71].

### 2.3. Safety Investigation

Although the effective dose is three orders of magnitude higher than antimalarial treatment dose, ARTs remains relatively much safer than other chemotherapy drugs used in clinics. However, it seems that ART derivatives can arouse potential embryotoxicity in experimental animals. For example, dihydroartemisinin can bring about abnormal embryonic phenotypes and promote early embryonic angiogenesis in zebrafish [72]. Preclinical studies have demonstrated that artesunate can induce congenital malformations in rodents [73,74]. In addition, in vitro experiments found that artemisinin, dihydroartemisinin, and artemether can lead to the death of neuronal cells, glial cells, and transformation neuronal cells by inducing the release of lactate dehydrogenase. In a study on the safety of artesunate conducted in healthy dogs, neither neurological nor cardiac toxicity was observed [75]; only hematological/gastrointestinal toxicity occurred, most of which were transient [76]. Nevertheless, what needs to be emphasized is that the toxicity of ARTs is its long-term effects, and unrelated to the short-term drug plasma concentration [23].

### 2.4. Clinical Experience

Although their excellent anticancer activities in vitro and in vivo are well documented, clinical data on artemisin in derivates are very limited. Several clinical observations for monomer analogs have been conducted. Studies have been conducted to explore artesunate and dihydroartemisinin for patients with metastatic breast cancer [25]. In small-scale clinical trials, the therapeutic effects of artesunate for colorectal cancer and uveal melanoma have been demonstrated. Recently, a united study group consisting of investigators from U.K., Germany, and Belgium completed a small clinical trial of oral artesunate for treatment of colorectal cancer, and they found that only one patient in the artesunate group (*n* = 9), but six patients in the placebo group (*n* = 11) developed recurrent colorectal cancer during a median follow up of 42 months [77]. Berger et al. treated two patients with uncontrollable advanced metastatic uveal melanoma with artesunateas the auxiliary standard chemotherapy, and found that one patient with stage IV survived more than 47 months, greatly exceeding the median survival of 2–5 months [78]. The treatment by the ultrasound-guided injection of artesunate into the tumor body in 18 cases of domestic primary advanced stage of liver cancer has been reported, and the results showed that, on the basis of comparison before and after treatment, the Alpha Fetal Protein (AFP) values, tumor size and echo, liver blood supply, etc., were improved significantly [79]. In the randomized controlled clinical trial on the combination of artesunate with vinorelbine and cisplatin for the treatment of advanced non-small cell lung cancer (NSCLC), it was found that the combination therapy group showed a significant improvement in the patient’s disease control rate and prolonged the time to progress [80]. In the Chinese folk, artemether injection was used for curing advanced breast cancer successfully. Artemether can also be used for treatment of pituitary macroadenoma, and the clinical symptoms of patients have been improved significantly after 12 months, and the impaired vision and hearing were gradually recovered [81].

## 3. Future Direction

### 3.1. Further Investigations on Mechanism of Action

The precise molecular targets of ARTs are still controversial and not fully elucidated yet, although its anticancer efficacy has been proved. Besides several previously revealed mechanisms, including increased oxidative stress, decreased proliferation, induction of apoptosis, and inhibition of angiogenesis, other pathways that ARTs may interfere with should be followed, such as more details on the iron-dependent hypothesis, anti-inflammatory effects on preventing tumor formation, synergistic or sensitizing mechanism of chemotherapy and radiotherapy, and ways to reverse multidrug resistance of tumor cells [82].

**Figure 3:**
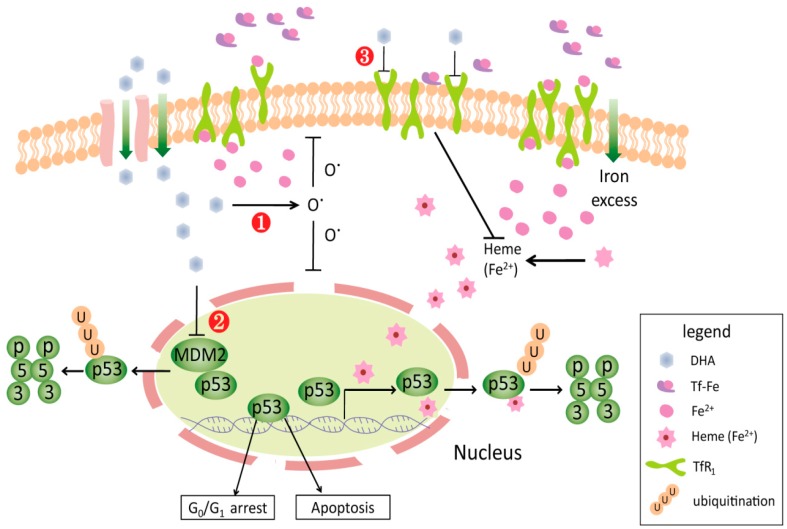
The multiple pathways of dihydroartemisinin (DHA) on tumor cells: ① killing effects of oxygen-free radicals on cell membrane and nucleus; ② binding to MDM2 in order to inhibit ubiquitination of p53; ③ down regulating levels of cell surface transferrin receptor (TfR1) in order to inhibit ubiquitination of p53.

The hypothesis of iron-dependent activation of artemisinin has been proposed and accepted, generally due to the fact that a high intracellular iron concentration was essential for continuous proliferation of tumor cells. More recently, a new mechanism has been identified that the excess iron polyporphyrin heme can bind to the p53 protein and trigger both nuclear export and cytosolic degradation of p53 [83]. Apparently, the major metabolite of ATRs, dihydroartemisinin, is a “dual- function” compound that inhibits ubiquitination of p53 through down regulating levels of cell surface transferrin receptor and binding to nuclear protein Murine Double Minute 2 (MDM2) [20,84] (Figure 3). The relevance of ROS-independent mechanisms, such as the anti-inflammatory mechanism, should be another area of great concern for ARTs. Numerous studies prove that ART and its derivatives have certain therapeutic effects on some acute and chronic inflammatory diseases. Although further explorations are necessary, anti-inflammatory effectiveness of ARTs probably plays an important role in the process of occurrence and progression of malignant tumors. Thirdly, it is necessary to carry out sensitive and/or resistant candidate genes screening and analysis of direct DNA damage induced by ART and the role of p53 status in genotoxicity. For instance, the analysis of gene expression profiling has identified artesunateas a novel topoisomerase inhibitor, which could lead to DNA damage [85]. It was also found that artesunate is resistant to genes related to antioxidation stress and EGFR, but not involved in multidrug resistance. Besides NF-κB, BCL-2, VEGF, survivin, andcaspase-3/8/9, ARTs were proposed to possibly participate in other multiple pathways including PI3K-Akt, T cell receptor, Toll-like receptor, transforming growth factor (TGF)-β, and insulin signaling pathways [86]. The research focusing on unveiling these supposed mechanisms will be greatly helpful to the development of targeted ART as well as its derivatives.

### 3.2. Exploration of an Efficient and Specific Drug Delivery System

Despite its promise as a broad-spectrum anticancer agent, ARTs still face some extra challenges in the design of formulations due to their poor solubility in water and oil and physicochemical instability [87]. Nanotargeted drug delivery systems (10–200 nm) have become the preferred orientation of antitumor therapy and could be developed for ART and its analogs. Drug-loaded nanostructured lipid carriers—such as lipid nanospheres or nanoliposomes—have been attempted [88,89,90]. It was demonstrated that artesunate nanoliposomes produced stronger efficacy on hepatocellular carcinoma as compared with free compound at the same concentration. The modified nanoparticles such as lacticacid-modified magnetic, lymphatics-homing peptide, or transferrin-modified nanoparticles maybe preferred because of ARTs’ characteristic anticancer mechanism. The submicron emulsion, which has also been investigated by Huang et al., possesses approximately 3-fold drug-loading capacity of dihydroartemisinin compared with the conventional oil-and-water (O/W) emulsion, and can be stored for up to 6 months stably, as well [91]. Another effective choice is the transferrin-ART graft-loaded functional nanoparticle due to the fact that its onset of endoperoxide bridge is iron-dependent. The graft copolymer can be peptides or proteins with excellent water-solubility and lipophilic properties. It was demonstrated that the uptake rates and antitumor effects of the graft were significantly higher than ARTs alone [92,93]. The researches on functional and especially multifunctional nanoparticles have increasingly become a hotspot in the field of nanotargeted drug delivery systems. The ART-loaded Fe_3_O_4_@C/Ag@mSiO_2_ mesopore can not only load a high capacity but can also reside in acidic compartments and demonstrate pH-responsive Fe^2+^ release to enhance cell killing as well [94,95] (Table 2). In addition, it should lay emphasis on development of oral formulations if ART is targeted to be an adjuvant agent in treatment of malignancies.

While the development of manufacturing techniques and application of nanoparticle drug carriers are becoming increasingly exciting and hopeful, there are many of challenges we have to face today, for instance biological barriers. As is known to all, the malignancies being developed within several biological barriers such as the blood–brain barrier, blood-pancreatic barrier, and blood-testis barrier are refractory irreversible and have generally resulted in poor prognosis. To date, there is very little knowledge about what and how nanoparticles pass through these biological barriers effectively [96]. It seems that ARTs are very promising chemotherapeutic agents in treatment of metastatic brain cancers due to their high effectiveness and low toxicity. Therefore, the innovative formulations being capable of effectively crossing the blood-brain barrier will be the main focus of the next step. There are some preclinical trials concerning nanoformulations for brain delivery, for example, multifunctional polymeric nanotheranostic system and resveratrol-loaded solid lipid nanoparticles [97,98]. Besides, the blood–pancreatic barrier also deserves equal attention, as it contributes to the high mortality rate of pancreas cancer.

### 3.3. Strategy in Clinical Application

Biomedical researches on ARTs have experienced revelation and innovation two periods [1] after the discovery of their anticancer activity. In the first period, ARTs should be prepared for application before entering the innovative period [99]. ART and its derivates satisfy all criteria for good chemotherapeutic agents, such as high specificity, high therapeutic index, and broad-spectrum effects against cancer cells but relatively low toxicity for normal cells. Thus, it is very promising that ARTs can eventually be developed into a new class of anticancer drugs in clinics. However, there are several aspects of concern that need further study, of which the clinical safety and effectiveness of the compound are indispensable.

Except the sporadic short-term neurotoxicity and embryotoxicity in animals, no evidence of toxicity has been found in patients with malaria, clinically. Even so, close attention should be paid to potential toxicity caused by multiple doses. So far, there is no long-term effect of administration, except for the data about continuous administration at a dose of 8 mg/kg/day for 40 weeks [100]. Thus, at higher doses, long-term toxicity to human organs such as hepatotoxicity, renal toxicity, and neurotoxicity should be investigated fully before entering clinical trials. Clinical trials of high quality are of crucial importance to a new drug being approved. The sporadic case reports or small-scale randomized controlled trials are well documented but remain unconvincing. The goal of clinical trials is to make a full evaluation on the safety and efficacy through choosing the surrogate endpoints (biomarkers). In order to decrease the late-stage attrition rate, it was the preference of the FDA in 2007 to undergo Phase 0 rather than Phase I, because the former greatly reduces the risks in human subjects [101]. In the specific protocols, the subjects in clinical trials of ARTs are the population of patients with tumors, and the surrogate endpoints with acceptable predictive values such as progression-Free-Survival (PFS)/time-to-progression (TTP)/risk ratio (RR) are usually used as the primary efficacy endpoints to predict the survival value. For those refractory patients with advanced cancer who had no response to the existing treatments or means, the single-arm trial design may be considered using the historical background data as the contrast.

Owing to its synergistic effects with other anticancer drugs and sensitizing action on tumor cells to chemotherapy and radiotherapy, there is likelihood that ARTs could be developed as chemotherapy synergists or radiotherapy sensitizers in the future [102]. Nevertheless, more work is needed to be done in clinic before that. For example, what kind of malignancy and chemotherapy agent can get the optimal results for the condition and with what drug combination? What is the optimal dose for the effect of enhanced sensitivity to radiotherapy? 

## 4. Conclusions

In summary, ARTs are now the focus in the field of the study on anticancer therapy due to the strong antitumor effects that have been proved to date, and they are expected to represent a new class of anticancer agents. However, further studies are needed to investigate their unknown mechanisms of action and an optimal drug delivery system, as well as determine sufficient safety and efficacy information from clinical trials.

## Figures and Tables

**Table 1:** Some representative repurposing agents for cancer therapy.

Agents	Purpose	Repurposing Cancer Types	References
Aspirin	Relieve pain, reduce fever, prevent blood from clotting	Gastrointestinal oncology, estrogen receptor-negative breast cancer	[2,3,4,5]
Metformin	Type 2 diabetes mellitus	Colorectal, breast, prostate colon, brain and non-small cell lung cancer	[6,7]
Thalidomide	Leprosy	Multiple myeloma, hepatocellular carcinoma	[8,9]
Cimitidine	Peptic ulcer	Colorectal cancer, melanoma, renal cell carcinoma, pancreatic carcinoma, Gastric carcinoma	[10,11]
Arsenic	Lung diseases and psoriasis	Leukemia	[12,13]

**Table 2:** Developed nanodrug delivery system for artemisinin and its derivatives (ARTs).

Compounds	Developed Formulations	Reference
Artemisinin	Magnetic nanoliposomes, adducts with human serum transferring, LyP-1 modification to polymeric micelles, multifunctional mesoporous nanoparticles	[89,92,93,94]
Artemether	Lipid nanospheres	[88]
Artesunate	Nanoliposomes, PLGA nanoparticles	[90,95]
Dihydroartemisinin	Nanoparticles-in-oil-in-water submicron emulsion	[91]

## References

[1] Boichuk S., Lee D.J., Mehalek K.R., Makielski K.R., Wozniak A., Seneviratne D.S., Korzeniewski N., Cuevas R., Parry J.A., Brown M.F. (2014). Unbiased compound screening identifies unexpected drug sensitivitie sand novel treatment options for gastrointestinal stromal tumors. Cancer. Res..

[2] Langley R.E., Rothwell P.M. (2014). Aspirin in gastrointestinal oncology: New data on an old friend. Curr. Opin. Oncol..

[3] Rothwell P.M., Price J.F., Fowkes F.G., Zanchetti A., Roncaglioni M.C., Tognoni G., Lee R., Belch J.F., Wilson M., Mehta Z. (2012). Short-term effects of daily aspirin on cancer incidence, mortality, and non-vascular death: Analysis of the time course of risks and benefits in 51 randomised controlled trials. Lancet.

[4] Algra A.M., Rothwell P.M. (2012). Effects of regular aspirin on long-term cancer incidence and metastasis: A systematic comparison of evidence from observational studies versus randomised trials. Lancet Oncol..

[5] Yiannakopoulou E.C. (2015). Aspirin and NSAIDs for breast cancer chemoprevention. Eur. J. Cancer Prev..

[6] Liu X., Chhipa R.R., Pooya S., Wortman M., Yachyshin S., Chow L.M., Kumar A., Zhou X., Sun Y., Quinn B. (2014). Discrete mechanisms of mTOR and cell cycle regulation by AMPK agonists independent of AMPK. Proc. Natl. Acad. Sci. USA.

[7] Kasznicki J., Sliwinska A., Drzewoski J. (2014). Metformin in cancer prevention and therapy. Ann. Transl. Med..

[8] Sonneveld P., Asselbergs E., Zweegman S., van der H.B., Kersten M.J., Vellenga E., van Marwijk-Kooy M., Broyl A., deWeerdt O., Lonergan S. (2015). Phase 2 study of carfilzomib, thalidomide, and dexamethasone as induction/consolidation therapy for newly diagnosed multiple myeloma. Blood.

[9] Wu J., Ng J., Christos P.J., Goldenberg A., Sparano J., Sung M.W., Hochster H.S., Muggia F.M. (2014). Chronic thalidomide and chemoembolization for hepatocellular carcinoma. Oncologist.

[10] Pantziarka P., Bouche G., Meheus L., Sukhatme V., Sukhatme V.P. (2014). Repurposing drugs in oncology (ReDO)-cimetidine as an anti-cancer agent. Ecancermedicalscience.

[11] Armitage J.O., Sidner R.D. (1979). Antitumour effect of cimetidine. Lancet.

[12] Zhang X.W., Yan X.J., Zhou Z.R., Yang F.F., Chen S.J., Chen Z. (2010). Arsenic trioxide controls the fate of the PML-RARα oncoprotein by directly binding PML. Science.

[13] Beauchamp E., Kosciuczuk E.M., Serrano R., Nanavati D., Swindell E.P., Viollet B., O’Halloran T.V., Altman J.K., Platanias L.C. (2015). Direct binding of arsenic trioxide to AMPK and generation of inhibitory effects on acute myeloid leukemia precursors. Mol. Cancer Ther..

[14] Collaboration Research Group for Qinghaosu (1977). A novel kind of sesquiterpene lactone-artemisinin. Chin. Sci. Bullet..

[15] Liu J.M., Ni M.Y., Zhou W.S. (1979). Structure and reaction of qinghaosu (Arteannuin). Acta. Chimica Sinica.

[16] Klayman D.L. (1985). Qinghaosu (artemisinin): An antimalarial drug from China. Science.

[17] Kakar Q., Sheikh S., Ahmed I., Khan M.A., Jamil M., ElMohammady H., Warsame M. (2016). Efficacy of artemisinin-based combination therapies for the treatment of falciparum malaria in Pakistan (2007–2015): In vivo response and *dhfr* and *dhps* mutations. Acta. Trop..

[18] Woerdenbag H.J., Moskal T.A., Pras N., Malingré T.M., El-Feraly F.S., Kampinga H.H., Konings A.W. (1993). Cytotoxicity of artemisinin-related endoperoxides to Ehrlich ascites tumor cells. J. Nat. Prod..

[19] Efferth T., Sauerbrey A., Olbrich A., Gebhart E., Rauch P., Weber H.O., Hengstler J.G., Halatsch M.E., Volm M., Tew K.D. (2003). Molecular modes of action of artesunate in tumor cell lines. Mol. Pharmacol..

[20] Hou J., Wang D., Zhang R., Wang H. (2008). Experimental therapy of hepatoma with artemisinin and its derivatives: In vitro and in vivo activity, chemosensitization, and mechanisms of action. Clin. Cancer Res..

[21] Chen T., Li M., Zhang R., Wang H. (2009). Dihydroartemisinin induces apoptosis and sensitizes human ovarian cancer cells to carboplatin therapy. J. Cell. Mol. Med..

[22] Wang Y.B., Hu Y., Li Z., Wang P., Xue Y.X., Yao Y.L., Yu B., Liu Y.H. (2013). Artemether combined with shRNA interference of vascular cell adhesion molecule-1 significantly inhibited the malignant biological behavior of human glioma cells. PLoS ONE.

[23] Alcntara D.D., Ribeiro H.F., Cardoso P.C., Araújo T.M., Burbano R.R., Guimarães A.C., Khayat A.S., Oliveira B.M. (2013). In vitro evaluation of the cytotoxic and genotoxic effects of artemether, an antimalarial drug, in a gastric cancer cell line (PG100). J. Appl. Toxicol..

[24] Azimi Mohamadabadi M., Hassan Z.M., Zavaran H.A., Gholamzad M., Noori S., Mahdavi M., Maroof H. (2013). Arteether exerts antitumor activity and reduces CD4+CD25+FOXP3+T-reg cells in vivo. Iran J. Immunol..

[25] Ericsson T., Blank A., von Hagens C., Ashton M., Abelö A. (2014). Population pharmacokinetics of artesunate and dihydroartemisinin during long-term oral administration of artesunate to patients with metastatic breast cancer. Eur. J. Clin. Pharmacol..

[26] Tu Y.Y. (2011). The discovery of artemisinin (qinghaosu) and gifts from Chinese medicine. Nat. Med..

[27] Zhu D.Y., Deng D.A., Zhang S.G., Xu R.S. (1984). Structure of artemisilactone. Acta Chimica Sinica.

[28] Gravett A.M., Liu W.M., Krishna S., Chan W.C., Haynes R.K., Wilson N.L., Dalgleish A.G. (2011). In vitro study of the anti-cancer effects of artemisone alone or in combination with other chemotherapeutic agents. Cancer Chemother Pharmacol..

[29] Yang H., Tan X.J. (2013). Research advance in antitumor activities of artemisinin and its derivatives. Zhong Guo Yi Xue Ke Xue Yuan Xue Bao.

[30] Chow L.M., Chan T.H. (2009). Novel classes of dimer antitumour drug candidates. Curr. Pharm. Des..

[31] Kong R., Jia G., Chen Z.X., Wang Y.W., Mu M., Wang S.J., Pan S.H., Gao Y., Jian H.C., Dong D.L. (2012). Dihydroartemisinin enhances Apo2L/TRAIL-mediated apoptosis in pancreatic cancer cells via ROS-mediated up-regulation of death receptor 5. PLoS ONE.

[32] Berdelle N., Nikolova T., Quiros S., Efferth T., Kaina B. (2011). Artesunateinduces oxidative DNA damage, sustained DNA double-strand breaks, and the ATM/ATR damage response in cancer cells. Mol. Cancer Ther..

[33] Mercer A.E., Copple I.M., Maggs J.L., O’Neill P.M., Park B.K. (2011). The role of heme and the mitochondrion in the chemical and molecular mechanisms of mammalian cell death induced by the artemisinin antimalarials. J. Biol. Chem..

[34] Efferth T. (2006). Molecular pharmacology and pharmacogenomics of artemisinin and its derivatives in cancer cells. Curr. Drug Targets.

[35] Nakase I., Gallis B., Takatani-Nakase T., Oh S., Lacoste E., Singh N.P., Goodlett D.R., Tanaka S., Futaki S., Lai H., Sasaki T. (2009). Transferrin receptor-dependent cytotoxicity of artemisinin-transferrin conjugates on prostate cancer cells and induction of apoptosis. Cancer Lett..

[36] Lu J.J., Meng L.H., Cai Y.J., Chen Q., Tong L.J., Lin L.P., Ding J. (2008). Dihydroartemisinin induces apoptosis in HL-60 leukemia cells dependent of iron and p38 mitogen-activated protein kinase activation but independent of reactive oxygen species. Cancer Biol. Ther..

[37] Chan H.W., Singh N.P., Lai H.C. (2013). Cytotoxicity of dihydroartemisinin toward Molt-4 cells attenuated by *N*-tert-butyl-α-phenylnitrone and deferoxamine. Anticancer Res..

[38] Jiang Z., Chai J., Chuang H.H., Li S., Wang T., Cheng Y., Chen W., Zhou D. (2012). Artesunate induces G0/G1 cell cycle arrest and iron-mediated mitochondrial apoptosis in A431 human epidermoid carcinoma cells. Anticancer Drugs..

[39] Chen G., Gong R., Shi X., Yang D., Zhang G., Lu A., Yue J., Bian Z. (2016). Halofuginone and artemisinin synergistically arrest cancer cells at the G1/G0 phase by upregulating p21Cip1 and p27Kip1. Oncotarget.

[40] Willoughby J.A., Sundar S.N., Cheung M., Tin A.S., Modiano J., Firestone G.L. (2009). Artemisinin blocks prostate cancer growth and cell cycle progression by disrupting Sp1 interactions with the cyclin-dependent kinase-4 (CDK4) promoter and inhibiting CDK4 gene expression. J. Biol. Chem..

[41] Chen K., Shou L.M., Lin F., Duan W.M., Wu M.Y., Xie X., Xie Y.F., Li W., Tao M. (2013). Artesunate induces G2/M cell cycle arrest through autophagy induction in breast cancer cells. Eur. J. Haematol..

[42] Steinbrück L., Pereira G., Efferth T. (2010). Effects of artesunate on cytokinesis and G0/M cell cycle progression of tumour cells and budding yeast. Cancer Geno. Proteom..

[43] Wang S.J., Gao Y., Chen H., Kong R., Jiang H.C., Pan S.H., Xue D.B., Bai X.W., Sun B. (2010). Dihydroartemisinin inactivates NF-κB and potentiates the anti-tumor effect of gemcitabine on pancreatic cancer both in vitro and in vivo. Cancer Lett..

[44] Cheng R., Li C., Li C., Wei L., Li L., Zhang Y., Yao Y., Gu X., Cai W., Yang Z. (2013). The artemisinin derivative artesunate inhibits corneal neovascularization by inducing ROS-dependent apoptosis in vascular endothelial cells. Investig. Ophthalmol. Vis. Sci..

[45] Li S., Xue F., Cheng Z., Yang X., Wang S., Geng F., Pan L. (2009). Effect ofartesunateon inhibiting proliferation and inducing apoptosis of SP2/0 myeloma cells through affecting NF-κB p65. Int. J. Hematol..

[46] Zeng Q.P., Zhang P.Z. (2011). Artesunatemitigates proliferation of tumor cells by alkylating heme-harboring nitric oxide synthase. Nitric Oxide.

[47] Yang N.D., Tan S.H., Ng S., Shi Y., Zhou J., Tan K.S., Wong W.S., Shen H.M. (2014). Artesunate induces cell death in human cancer cells via enhancing lysosomal function and lysosomal degradation of ferritin. J. Biol. Chem..

[48] Zhou C., Pan W., Wang X.P., Chen T.S. (2012). Artesunate induces apoptosis via a Bak-mediated caspase-independent intrinsic pathway in human lung adenocarcinoma cells. J. Cell. Physiol..

[49] Hamacher-Brady A., Stein H.A., Turschner S., Toegel I., Mora R., Jennewein N., Efferth T., Eils R., Brady N.R. (2011). Artesunate activates mitochondrial apoptosis in breast cancer cells via iron-catalyzed lysosomal reactive oxygen species production. J. Biol. Chem..

[50] Ji Y., Zhang Y.C., Pei L.B., Shi L.L., Yan J.L., Ma X.H. (2011). Anti-tumor effects of dihydroartemisinin on human osteosarcoma. Mol. Cell. Biochem..

[51] Holien T., Olsen O.E., Misund K., Hella H., Waage A., Rø T.B., Sundan A. (2013). Lymphoma and myeloma cells are highly sensitive to growth arrest and apoptosis induced by artesunate. Eur. J. Haematol..

[52] Jiao Y., Ge C.M., Meng Q.H., Cao J.P., Tong J., Fan S.J. (2007). Dihydroartemisinin is an inhibitor of ovarian cancer cell growth. Acta Pharmacol. Sin..

[53] Chen H., Sun B., Wang S., Pan S., Gao Y., Bai X., Xue D. (2010). Growth inhibitory effects of dihydroartemisinin on pancreatic cancer cells: Involvement of cell cycle arrest and inactivation of nuclear factor-κB. J. Cancer Res. Clin. Oncol..

[54] Lu J.J., Meng L.H., Shankavaram U.T., Zhu C.H., Tong L.J., Chen G., Lin L.P., Weinstein J.N., Ding J. (2010). Dihydroartemisinin accelerates c-MYC oncoprotein degradation and induces apoptosis in c-MYC-overexpressing tumor cells. Biochem. Pharmacol..

[55] Blazquez A.G., Fernandez-Dolon M., Sanchez-Vicente L., Maestre A.D., Gomez-San M.A.B., Alvarez M., Serrano M.A., Jansen H., Efferth T., Marin J.J. (2013). Novel artemisinin derivatives with potential usefulness against liver/colon cancer and viral hepatitis. Bioorg. Med. Chem..

[56] Efferth T., Romero M.R., Wolf D.G., Stamminger T., Marin J.J., Marschall M. (2008). The antiviral activities of artemisinin and artesunate. Clin. Infect. Dis..

[57] Romero M.R., Efferth T., Serrano M.A., Castaño B., Macias R.I., Briz O., Marin J.J. (2005). Effect of artemisinin/artesunate as inhibitors of hepatitis B virus production in an “in vitro” replicative system. Antiviral. Res..

[58] Ooko E., Saeed M.E., Kadioglu O., Sarvi S., Colak M., Elmasaoudi K., Janah R., Greten H.J., Efferth T. (2015). Artemisinin derivatives induce iron-dependent cell death (ferroptosis) in tumor cells. Phytomedicine.

[59] Mu D., Chen W., Yu B., Zhang C., Zhang Y., Qi H. (2007). Calcium and survivin are involved in the induction of apoptosis by dihydroartemisinin in human lung cancer SPC-A-1 cells. Methods Find Exp. Clin. Pharmacol..

[60] Mu D., Zhang W., Chu D., Liu T., Xie Y., Fu E., Jin F. (2008). The role of calcium, P38 MAPK in dihydroartemisinin-induced apoptosis of lung cancer PC-14 cells. Cancer Chemother Pharmacol..

[61] Jia G., Kong R., Ma Z.B., Han B., Wang Y.W., Pan S.H., Li Y.H., Sun B. (2014). The activation of c-Jun NH_2_-terminal kinase is required for dihydroartemisinin-induced autophagy in pancreatic cancer cells. J. Exp. Clin. Cancer Res..

[62] Njokah M.J., Kang’ethe J.N., Kinyua J., Kariuki D., Kimani F.T. (2016). In vitro selection of *Plasmodium falciparum Pfcrt* and *Pfmdr1* variants by artemisinin. Malar. J..

[63] Wang S.J., Sun B., Cheng Z.X., Zhou H.X., Gao Y., Kong R., Chen H., Jiang H.C., Pan S.H., Xu D.B. (2011). Dihydroartemisin in inhibits angiogenesis in pancreatic cancer by targeting the NF-κB pathway. Cancer Chemother. Pharmacol..

[64] Dong F., Zhou X., Li C., Yan S., Deng X., Cao Z., Li L., Tang B., Allen T.D., Liu J. (2014). Dihydroartemisin in targets VEGFR2 via the NF-κB pathway in endothelial cells to inhibit angiogenesis. Cancer Biol. Ther..

[65] Guo L., Dong F., Hou Y., Cai W., Zhou X., Huang A.L., Yang M., Allen T.D., Liu J. (2014). Dihydroartemisin in inhibits vascular endothelial growth factor-induced endothelial cell migration by a p38 mitogen-activated protein kinase- independent pathway. Exp. Ther. Med..

[66] Saeed M.E., Kadioglu O., Seo E.J., Greten H.J., Brenk R., Efferth T. (2015). Quantitative structure-activity relationship and molecular docking of artemisinin derivatives to vascular endothelial growth factor receptor 1. Anticancer Res..

[67] Chen H., Shi L., Yang X., Li S., Guo X., Pan L. (2010). Artesunate inhibiting angiogenesis induced by human myeloma RPMI8226 cells. Int. J. Hematol..

[68] Konkimalla V.B., McCubrey J.A., Efferth T. (2009). The role of downstream signaling pathways of the epidermal growth factor receptor for artesunate’s activity in cancer cells. Curr. Cancer Drug Targets..

[69] Ma H., Yao Q., Zhang A.M., Lin S., Wang X.X., Wu L., Sun J.G., Chen Z.T. (2011). The effects of artesunate on the expression of EGFR and ABCG2 in A549 human lung cancer cells and a xenograft model. Molecules.

[70] Li L.N., Zhang H.D., Yuan S.J., Yang D.X., Wang L., Sun Z.X. (2008). Differential sensitivity of colorectal cancer cell lines to artesunate is associated with expression of β-catenin and E-cadherin. Eur. J. Pharmacol..

[71] Krusche B., Arend J., Efferth T. (2013). Synergistic inhibition of angiogenesis by artesunate and captopril in vitro and in vivo. Evid. Based Compl. Alternat. Med..

[72] Ba Q., Duan J., Tian J.Q., Wang Z.L., Chen T., Li X.G., Wu S.J., Xiang L., Li J.Q., Chu R.A. (2013). Dihydroartemisinin promotes angiogenesis during the early embryonic development of zebrafish. Acta Pharmacol. Sin..

[73] Li Q., Weina P.J. (2009). Severe embryotoxicity of artemisinin derivatives in experimental animals, but possibly safe in pregnant women. Molecules.

[74] Gomes C., Boareto A.C., Dalsenter P.R. (2016). Clinical and non-clinical safety of artemisinin derivatives in pregnancy. Reprod. Toxicol..

[75] Rutteman G.R., Erich S.A., Mol J.A., Spee B., Grinwis G.C., Fleckenstein L., London C.A., Efferth T. (2013). Safety and efficacy field study of artesunate for dogs with non-resectable tumours. Anticancer Res..

[76] Schmuck G., Roehrdanz E., Haynes R.K., Kahl R. (2002). Neurotoxic mode of action of artemisinin. Antimicrob. Agents Chemother..

[77] Krishna S., Ganapathi S., Ster I.C., Saeed M.E., Cowan M., Finlayson C., Kovacsevics H., Jansen H., Kremsner P.G., Efferth T. (2015). A Randomised, Double Blind, Placebo-Controlled Pilot Study of Oral Artesunate Therapy for Colorectal Cancer. EBioMedicine.

[78] Berger T.G., Dieckmann D., Efferth T., Schultz E.S., Funk J.O., Baur A., Schuler G. (2005). Artesunate in the treatment of metastatic uveal melanoma-first experiences. Oncol. Rep..

[79] Zhang Y., Zhang Y., Ren J. (2007). Application Value of Interventional Ultrasound in Treatment of Primary Hepatic. Mod. Diag. Treat..

[80] Zhang Z.Y., Yu S.Q., Miao L.Y., Huang X.Y., Zhang X.P., Zhu Y.P., Xia X.H., Li D.Q. (2008). Artesunate combined with vinorelbine plus cisplatin in treatment of advanced non-small cell lung cancer: A randomized controlled trial. Zhong Xi Yi Jie He Xue Bao.

[81] Singh N.P., Panwar V.K. (2006). Case report of a pituitary macro adenoma treated with artemether. Integr. Cancer Ther..

[82] Huang Y., Li H., Peng D., Wang Y., Ren Q., Guo Y. (2016). The production and exportation of artemisinin-derived drugs in China: current status and existing challenges. Malar. J..

[83] Shen J., Sheng X., Chang Z., Wu Q., Wang S., Xuan Z., Li D., Wu Y., Shang Y., Kong X. (2014). Iron metabolism regulates p53 signaling through direct heme-p53 interaction and modulation of p53 localization, stability, and function. Cell Rep..

[84] Ba Q., Zhou N., Duan J., Chen T., Hao M., Yang X., Li J., Yin J., Chu R., Wang H. (2012). Dihydroartemisinin exerts its anticancer activity through depleting cellular iron via transferrin receptor-1. PLoS ONE.

[85] Li P.C., Lam E., Roos W.P., Zdzienicka M.Z., Kaina B., Efferth T. (2008). Artesunate derived from traditional Chinese medicine induces DNA damage and repair. Cancer Res..

[86] Huang C., Ba Q., Yue Q., Li J., Li J., Chu R., Wang H. (2013). Artemisinin rewires the protein interaction network in cancer cells: Network analysis, pathway identification, and target prediction. Mol. Biosyst..

[87] Ochiuz L., Grigoras C., Popa M., Stoleriu I., Munteanu C., Timofte D., Profire L., Grigoras A.G. (2016). Alendronate-Loaded Modified Drug Delivery Lipid Particles Intended for Improved Oral and Topical Administration. Molecules.

[88] Eltayeb S.E., Su Z., Shi Y., Li S., Xiao Y., Ping Q. (2013). Preparation and optimization of transferrin-modified- artemether lipid nanospheres based on the orthogonal design of emulsion formulation and physically electrostatic adsorption. Int. J. Pharm..

[89] Gharib A., Faezizadeh Z., Mesbah-Namin S.A., Saravani R. (2014). Preparation, characterization and in vitro efficacy of magnetic nanoliposomes containing the artemisinin and transferrin. DARU J. Pharm. Sci..

[90] Jin M., Shen X., Zhao C., Qin X., Liu H., Huang L., Qiu Z., Liu Y. (2013). In vivo study of effects of artesunate nanoliposomes on human hepatocellular carcinoma xenografts in nude mice. Drug Deliv..

[91] Wang S., Wang H., Liang W., Huang Y. (2012). An injectable hybrid nanoparticle-in-oil-in-water submicron emulsion for improved delivery of poorly soluble drugs. Nanoscale Res. Lett..

[92] Yang Y., Zhang X., Wang X., Zhao X., Ren T., Wang F., Yu B. (2014). Enhanced delivery of artemisinin and its analogues to cancer cells by their adducts with human serum transferrin. Int. J. Pharm..

[93] Wang Z., Yu Y., Ma J., Zhang H., Zhang H., Wang X., Wang J., Zhang X., Zhang Q. (2012). LyP-1 modification to enhance delivery of artemisinin or fluorescent probe loaded polymeric micelles to highly metastatic tumor and its lymphatics. Mol. Pharm..

[94] Chen J., Guo Z., Wang H.B., Zhou J.J., Zhang W.J., Chen Q.W. (2014). Multifunctional mesoporous nanoparticles as pH-responsive Fe^2+^ reservoirs and artemisinin vehicles for synergistic inhibition of tumor growth. Biomaterials.

[95] Nguyen H.T., Tran T.H., Kim J.O., Yong C.S., Nguyen C.N. (2015). Enhancing the in vitro anti-cancer efficacy of artesunate by loading into poly-d,l-lactide-co-glycolide (PLGA) nanoparticles. Arch. Pharm. Res..

[96] McCarthy D.J., Malhotra M., O’Mahony A.M., Cryan J.F., O’Driscoll C.M. (2015). Nanoparticlesand the Blood-BrainBarrier: Advancing from in-Vitro Models towards Therapeutic Significance. Pharm. Res..

[97] Li J., Cai P., Shalviri A., Henderson J.T., He C., Foltz W.D., Prasad P., Brodersen P.M., Chen Y., DaCosta R. (2014). A multifunctional polymeric nanotheranostic system delivers doxorubicin and imaging agents across the blood-brain barrier targeting brain metastases of breast cancer. ACS Nano..

[98] Jose S., Anju S.S., Cinu T.A., Aleykutty N.A., Thomas S., Souto E.B. (2014). In vivo pharmacokinetics and biodistribution of resveratrol-loaded solid lipid nanoparticles for brain delivery. Int. J. Pharm..

[99] Goldstein J.L. (2007). Creation and revelation: Two different routes to advancement in the biomedical sciences. Nat. Med..

[100] Lai H.C., Singh N.P., Sasaki T. (2013). Development of artemisinin compounds for cancer treatment. Investig. New Drugs..

[101] Gupta U.C., Bhatia S., Garg A., Sharma A., Choudhary V. (2011). Phase 0 clinical trials in oncology new drug development. Perspect. Clin. Res..

[102] Zhao Y., Jiang W., Li B., Yao Q., Dong J., Cen Y., Pan X., Li J., Zheng J., Pang X. (2011). Artesunate enhances radio sensitivity of human non-small cell lung cancer A549 cells via increasing NO production to induce cell cycle arrest at G2/M phase. Int. Immunopharmacol..

